# Exploring motivations behind pollution-mask use in a sample of young adults in urban China

**DOI:** 10.1186/s12992-018-0441-y

**Published:** 2018-12-04

**Authors:** Francesca Valeria Hansstein, Fabián Echegaray

**Affiliations:** 1 School of Public Economics and Administration, Shanghai University of Finance and Economics, 111 Wuchuan Road, Yangpu District, Shanghai, 200433 China; 2 Market Analysis, Rua Felix Kleis, 23, Florianópolis, SC 88035330 Brazil

**Keywords:** Pollution mask, Air pollution, Health prevention, Health promotion, Theory of planned behaviour, China

## Abstract

**Background:**

Wearing a pollution mask is an effective, practical, and economic way to prevent the inhalation of dangerous particulate matter (PM). However, it is not uncommon to observe negligence in adopting such behaviour, and this especially among young segments of the population. Using the Theory of Planned Behaviour (TPB) as conceptual framework, this study explores the role of socio-cognitive factors that affect the decision of wearing a pollution mask in the context of young educated people. This is done by selecting a sample of college students in urban China, a country that has seen air quality as one of the major challenges in the last decades. While young urban college students might be expected to be receptive to standard attempts to be influenced through reason-based cognitive stimuli, it is often found that this is not the case. The empirical analysis was articulated it in two steps. Structural Equation Modelling (SEM) was first used to examine the relationships among the conceptual constructs derived from the TPB conceptual model, and second Step-Wise Ordinary Least Squares Regressions (SWOLS) were employed to observe the partial effect played by each item on the decision to wear a mask.

**Results:**

Results show that, while reason-based stimuli play a role, attitude, social norm, and self-efficacy were the most important predictors of the behavioural intention (*p* < 0.01). The role of past behaviour was also acknowledged as strongly associated with the dependent variable (*p* < 0.01). Overall, the likelihood of wearing a pollution mask increases with the importance of others socio-cognitive and psychological factors, which could help understand behavioural biases, and explain the relative role of several mechanisms behind the decision to wear a mask.

**Conclusions:**

While tackling pollution requires multiple and synergic approaches, encouraging self-prevention using pollution mask is a simple and effective action, implementable at negligible costs. Resistance among younger, well-educated cohorts to wear masks can be overcome by stressing the social desirability of action and the sense of empowerment derived from its usage. This study has the potential to inform policies aimed at changing suboptimal behavioural attitudes by identifying triggers for change, and it could serve in improving the tailoring of health promotion messages aimed at nudging healthy behaviour.

**Electronic supplementary material:**

The online version of this article (10.1186/s12992-018-0441-y) contains supplementary material, which is available to authorized users.

## Background

Since the beginning of its rapid economic development, China started experiencing one of the worst air pollution emergencies in the world’s history, affecting especially urban areas and that has become one of the leading threats to public health [1, 2]. According to the Yale and Columbia University’s Environmental Performance Index, and with the exception of a handful of poor nations, China’s pollution trails all developed and developing countries, and ranked second to last in air quality [3]. Particulate matter of inhalable and respirable size fractions (i.e. PM10 and PM2.5) represents the air pollutant of greatest health concern in urban China [4, 5] Due to their microscopic size particles of this nature easily enter the human respiratory system, contributing to respiratory illnesses.. Moreover, prolonged exposure to air pollution has negative effects on cognitive abilities later in life [6], whereas expecting mothers can experience a higher frequency of pregnancy complications related to excessive exposure to PM [7]. In terms of tangible health effects caused by pollution, low-educated and elderly females are the most vulnerable population segment [8]. In addition to the negative effects on individual health, air pollution represents a direct drag on public resources by rising costs for the health system, and an indirect hindrance to the economic and social order by incentivizing mass emigration.

In 2016, the Chinese government has launched a five-year plan to radically tighten air pollution and initiated a process of green growth and development. During the Asia-Pacific Economic Cooperation in 2014, the Chinese government successfully implemented rigorous emission reduction initiatives to lower air pollution levels in the city of Beijing [9, 10]. The experiment achieved excellent results, demonstrating that pollution can be tackled with adequate policy measures. However, the positive effects of these measures on health are likely to emerge in the long-run. Far less clear is government success in redressing pollution effects upon individuals and in affecting individuals to responsibly handle preventive health measures when dealing with pollution. The Chinese government took in fact several initiatives and leveraged on mass media dissemination to encourage prevention against pollution. These included, for example, the daily reporting of environmental conditions, the dissemination of scientific knowledge about the adverse effects of pollution exposure, advising sensitive groups like the elderly, children, or people with respiratory diseases against spending long periods outdoors and several others.

Among the solutions to reduce exposure to air pollution are: (a) staying indoors with air purifiers, (b) avoiding physical activity during severely polluted days, and (c) reducing frying of food and smoking at home. One of the most effective alternatives to reducing personal exposure to particulate matter, specifically outdoors, is also wearing a pollution mask. The promotion of pollution mask wearing has been one of the core government-initiated programs to guide individual responses to pollution exposures. However, in spite of public promotion, being a practical and straightforward form of self-prevention, as well as one of the cheapest, it is found that a large number of individuals do not wear a pollution mask during polluted days. This resistance is stronger among the youngest and best educated people of big cities in the country, who interestingly are also the ones more easily targeted by those initiatives of government officials aiming at a wide dissemination of information, warnings, and best practices to protect against pollution [11]. This is puzzling to some extent, and suggests that, because of the extreme cost effectiveness and accessibility to masks, most of the impediments to adopt such a healthy habit are likely to be found in behaviours informed and conditioned by individual attitudes and social pressure. Moreover, the efficacy of facemasks in preventing the inhalation of PM has been successfully tested, and previous research has shown that there are immediate positive outcomes on blood pressure and heart rate [12]. Finally, masks are a useful tool to protect individuals from the transmission of acute respiratory infections and pandemic influenza [13]. However, to our knowledge, there is no study documenting the reasons for the lack of adherence to mask-wearing registered especially among the youngest population. This paper aims at filling this gap, and provides empirical evidence gauging the relative contribution of extra-cognition stimuli in driving accurate preventive behaviours regarding pollution facemask use among young, well-educated, and urban Chinese population. This is the population segment with the greatest resistance to adopt risk-averse health practices when dealing with pollution.

To conduct the empirical investigation, the Theory of Planned Behaviour (TPB) was applied to determine the role that socio-cognitive factors play on the decision of wearing a pollution mask among a sample of Chinese students. At this scope, two quantitative analyses were employed. The first is based on Structural Equation Modelling (SEM); this methodology was used to examine the relationships among conceptual constructs measuring the intention to wear a mask. The second model used Step-Wise Ordinary Least Squares regressions (SWOLS) to estimate the marginal effect of each item, identified at the SEM stage, on mask wearing intention.

## Methods

### Theoretical framework

Beyond simple utility-based rational models of behaviours, the literature acknowledges a diversity of reasons behind the use of facemasks. Awareness of adverse consequences towards future health, as well as raw fears for personal wellbeing, are among the more emotional forces reported by the literature [14, 15] to trigger the use of facemasks. Likewise, environmental conditions also seem to drive behaviours, given that a 100-point increase in the Air Quality Index (AQI) has been linked to an increase in the purchase of facemasks by 54.5% and anti-PM2.5 masks by 70.6%; in fact, the search of the word “mask” (口罩 in Chinese) on internet search engines escalated in days with a high the AQI [16].

Emotional reactions are fed by perceived effects of air pollution which implies that individuals first and foremost develop attitudes based on perceptions and assess the extent to which the target behaviour is within their reach. Additionally, the elasticity of demand for face mask suggests that affordability issues do not impair individuals from adopting the behaviour if they want so, in other words, they don’t feel disempowered to perform the action.

Given the shortcomings of cognitive modelling of action for facemask use, socio-psychological stimuli should be recognized as influential like how individuals perceive the legitimacy of performing the target behaviour, their ability and capacity to do so, and how this target behaviour is associated with positive feelings. The Theory of Planned Behaviour (TPB) [17] assumes that individuals make choices based on weighting the possibilities and obstacles to enact the expected action, and thus building a sense of confidence in actually achieving desired results, their broader feelings towards the action at stake, and their surrounding social context. Other forces may be at play as well such as their past reactions to similar situations. Previous research had proven the validity of the theory-based psychological models in explaining the intention to take preventive measure for limiting the health effects of air pollution [18, 19]. Our hypothesis is that other influences like the prevailing social norms, inertial reactions replicating past behaviours, and the perceived feasibility of implementing new behaviours have also a role in making sense of unhealthy personal choices.

**Fig. 1:**
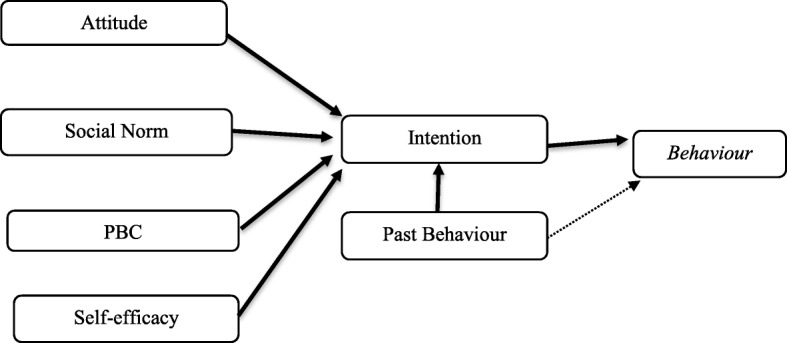
The theory of Planned Behaviour (TPB)

TPB proposes that behavioural intention is determined by attitude towards the behaviour, subjective or social norm, and perceived behavioural control (PBC). Three types of perceptions influence involvement with the target action: (a) the perceptions of how socially acceptable or mandatory the intended behaviour among individual’s salient reference groups is (i.e., social norms); (b) the perceptions of how easy or hard it is to set such behaviour in motion, in other words, the perceived capacity of overcoming barriers – or to take advantage of facilitators – to engage the behaviour (i.e., PBC); (c) and lastly the perceptions of how likeable or unwanted the intended behaviour pictured in people’s minds is, mostly based on the perceived relevance and payoffs of the consequences of enacting such behaviour (attitudes) [20, 21]. TPB is one of the most widely applied theories for health prevention, while also contributing to the understanding and overcoming of behavioural-change resistance [22] (Fig. 1).

### Design and statistical analysis

This study employed an exploratory sequential mixed methods research design. A preliminary qualitative phase on a small sample of the target population - to elicit the salient beliefs towards the behaviour and to build the questionnaire - was followed by data collection and the quantitative analysis. Qualitative research also enabled the validation of how expressions of social norms, perceived behavioural control and attitudes towards mask use actually play a role in students’ calculus of mask use, together with the recognition of other forces influencing choice such as past behaviours, that is, the influence of inertial behaviour. To evaluate the appropriateness of the questionnaire items, a principal component factor analysis was first performed, and, as a second step, the model constructs were built using the mean score method. In particular, for each respondent, one score was calculated as the mean of the answers to the items. The main difference between factor-score and mean-score method is that mean score assumes that each item is equally important to the concept being measured, while factor score does not. In our analysis, the assumption was met so constructs were calculated using the mean score method. In addition, the interpretation of the mean score is straightforward because each construct has the original scale used for the items. Secondly, the mean score manages missing values more efficiently [23].

The dependent variable of this study was measured based on respondents’ degree of agreement towards seven different behavioural reactions all connected to wearing a pollution mask when confronted with a very specific situation which is “*if the AQI is higher than 200 and I have to stay outdoors for more than one hour”.* It is fair to acknowledge that the average AQI during fieldwork did not reach 200. Those seven reactions related to 1) expecting, 2) wanting, 3) choosing, 4) intending, 5) preferring, 6) suitability, and 7) future commitment to use a mask. A seven-point Likert scale was used to identify the level of agreement with each reaction. To account for the nominal heterogeneity behind construct’s content, the measure included a variety of states which demand different intensity of individual commitment to action. All reactions cluster together yielding a high Cronbach’s alpha reliability index of 0.94. Accordingly, the intention to behave construct is thereby robustly measured. The need for specifying in greater detail the exact context of performing the action (that is, reaching AQI higher than 200) derived from the former round of qualitative research which clearly suggested that a context-free assessment of pollution-related preventive behaviours would have led to a problematic unbinding of the action, which in turn stimulated overstatements about individual unwillingness to adopt health-conscious practices. The other measures of the standard TPB model also followed a multi-item construct composition. Attitudes towards the behaviour aims to measure the perceived consequences and value of performing the action and thus was measured using a seven-point Likert scale that grasped the agreement with the idea of wearing a mask as 1) necessary, 2) effective, 3) beneficial, and 4) useful. Except for the latter item which loads at 0.57, all other items have a high correlation to the latent trait, and therefore contribute to yielding a valid and reliable construct. Cronbach’s alpha for the construct was 0.76. Social norms capture the surrounding environment of pressures and conformity to other people’s expectations with regards to using a facemask against pollution, thus it was measured as the perceived reaction of four groups: 1) parents, 2) friends, 3) schoolmates, and 4) roommates to that behaviour, using a seven-point Likert agreement scale. The inter-item correlation was sufficiently high to yield a highly satisfactory Cronbach alpha coefficient of 0.80. Our study also included questions for Perceived Behavioural Control (PBC) and self-efficacy. PBC relates to the possibilities that action could be performed in practical terms and the degree of confidence that initiatives taken will amount to the desired behaviour and the expected outcomes from it. Self -efficacy is a key factor in explaining the adoption of health-related behaviours and is in fact always included in many health behavioural models [24]. Although there are criticisms in the literature towards the measurement redundancy of PBC and self-efficacy [25] constructs were kept separated in this research following the approach of similar studies examining health behaviours or college students [26, 27]. These studies effectively demonstrated the separability of the two concepts: one side there is the perceived confidence in individual ability to achieve the behavioural outcome, on the other the belief that the outcome can successfully be influenced by one’s own effort. From an empirical point of view, in our study, while PBC measured the role of objective obstacles (like accessibility and affordability), the question on self-efficacy was intended to measure the general subjective evaluation on oneself capacity to succeed in performing the specific task, which is not just simply wearing a mask, but also adopting self-preventive behaviours. Buying an anti-pollution mask on campus is a relatively easy task - therefore unrelated to individual self-efficacy. It seems reasonable to acknowledge that a simple task like buying a mask cannot necessarily affect the target behaviour whereas the belief that wearing a mask can successfully protect ones’ health may drive the behavioural intention. This was also confirmed by the low correlation between the PBC and self-efficacy items (*r* = 0.32).

Lastly, in order to better specify the model and given its weight in shaping health prevention choices past behaviour was also included in the analysis. This choice integrates the role of habits and, mainly, prior experience. A meta-analysis research on health behavioural determinants has shown how the addition of past behaviour increased by 19% the variance explained by the core constructs. When possible, the inclusion of past behaviour is recommended for a better model’s specification and more precision in coefficient estimation. It captures the essential role of habit in shaping the behavioural intention and it has also found to attenuate the role of other attitude [28]. Past behaviour was measured asking first: *In the last year (s), did you wear a pollution mask during high polluted days?* And then for those who answered positively, the variable’s frequency was recoded to be consistent with the other constructs (*how often, from never to always*).

### Target population

The questionnaire was administered in Chinese and was translated into English for dataset building and data analysis purposes (see Additional file 1 for more information). Survey data were collected between Nov 27 and Nov 30, 2015, and a total of 407 respondents participated in the study. Of these, 386 completed the questionnaire, thus leading to a completion rate of 95%. During the days of data collection, the average AQI was 121.5 (respectively 85, 112, 110 and 179). For this average AQI level government indicates that slight irritations may occur; individuals with breathing or heart problems should reduce outdoor activities.

This study employed a convenient sample, and participants were recruited among students living on campus in Shanghai. Although this choice can introduce a bias in the generalization of the results, the primary goal of this study is to study the factors affecting the behaviour within an educated and urban sample of Chinese young individuals. A more detailed discussion about the use of convenience samples can be found in the discussion. Informed consent was obtained from all participants included in the study. Interviewers were properly trained on how to conduct the survey and responses were collected via self-administered questionnaires. Also, the interviewers were reachable anytime in case the respondents needed some clarification. This procedure left respondents the privacy to reflect and answer the questions, and, at the same time, guaranteed the comprehension of the items in case they needed clarifications.

## Results

Table 1 reports a detailed description of the variables used to calculate the model constructs, together with the Cronbach’s alpha and factor loadings for each item. Cronbach’s alpha is a measure of internal consistency and indicates whether a measure is one-dimensional. The minimum standard accepted threshold is 0.7. Similarly, factor loadings indicate how much variability of each item is correlated to the latent construct (in this case, Factor 1), with values above 0.63 it is considered very good [29].

**Table 1:** Measures of the items used to build the TPB constructs

Construct	Measures	Factor loadings
Intention alpha = 0.94	If the AQI is higher than 200 and if I have to stay outdoor for more than 1 hour consecutively…	
	I expect to wear a pollution mask	0.87
	I want to wear a pollution mask	0.87
	I intend to wear a pollution mask	0.92
	I choose to wear	0.90
	I will wear a pollution mask	0.88
	I would be better wearing a pollution mask	0.72
	I prefer wearing a pollution mask	0.85
Attitude Alpha = 0.76	Wearing a pollution mask is…	
	Necessary	0.83
	Effective	0.87
	Beneficial	0.76
	Useful	0.57
Social Norm Alpha = 0.80	Do you think that the following individual or groups would approve or disapprove that you wear a pollution mask…?	
	My parents	0.70
	My friends	0.84
	My schoolmates	0.79
	My roommates	0.81
Perceived Behavioural Control (PBC) Alpha = 0.80	How likely are you going to face the following situations if you wear a pollution mask during a day with AQI higher than 200 if I have to stay outdoor for more than 1 h?	
	I will find a shop where to buy a pollution masks	0.84
	I can find high-quality pollution masks	0.90
	I will be recommended a specific mask brand	0.80

Descriptive statistics were first calculated to determine the distributions of the core model constructs. Structural equation models in the form of path analyses were performed to evaluate the relative impact of each construct on the intention to wear a pollution mask. The fit of the model was assessed through the examination of these fit indices: chi2 test, the comparative fix index (CFI) and the root mean square error of approximation (RMSEA).

Table 2 presents the descriptive statistics of the core variables. Overall, 53% of respondents were females, and 47% males; 77% came from urban areas, and 33% had a rural origin. Respondents were on average 22 years old. Although the mean value of the construct intention was quite high (5.13), the average of the frequency with which respondents used a facemask in the past was quite low (3.46). Subjective norm, attitude, self-efficacy, and PBC were also relatively high.

**Table 2:** Sample descriptive statistics (*n* = 386)

Variable	Mean ± SD	Min	Max
Gender (female = 1)	.53 ± .49	0	1
Residency (urban = 1)	.77 ± .41	0	1
Age (years)	22.5 ± 2.21	18	38
Intention	5.13 ± 1.58	1	7
Attitude	4.79 ± 1.28	1	7
Social Norm	5.63 ± .97	1	7
PBC	4.56 ± 1.5	1	7
Self-efficacy	4.86 ± 1.92	1	7
Past behaviour	3.46 ± 2.03	1	7

**Fig. 2:**
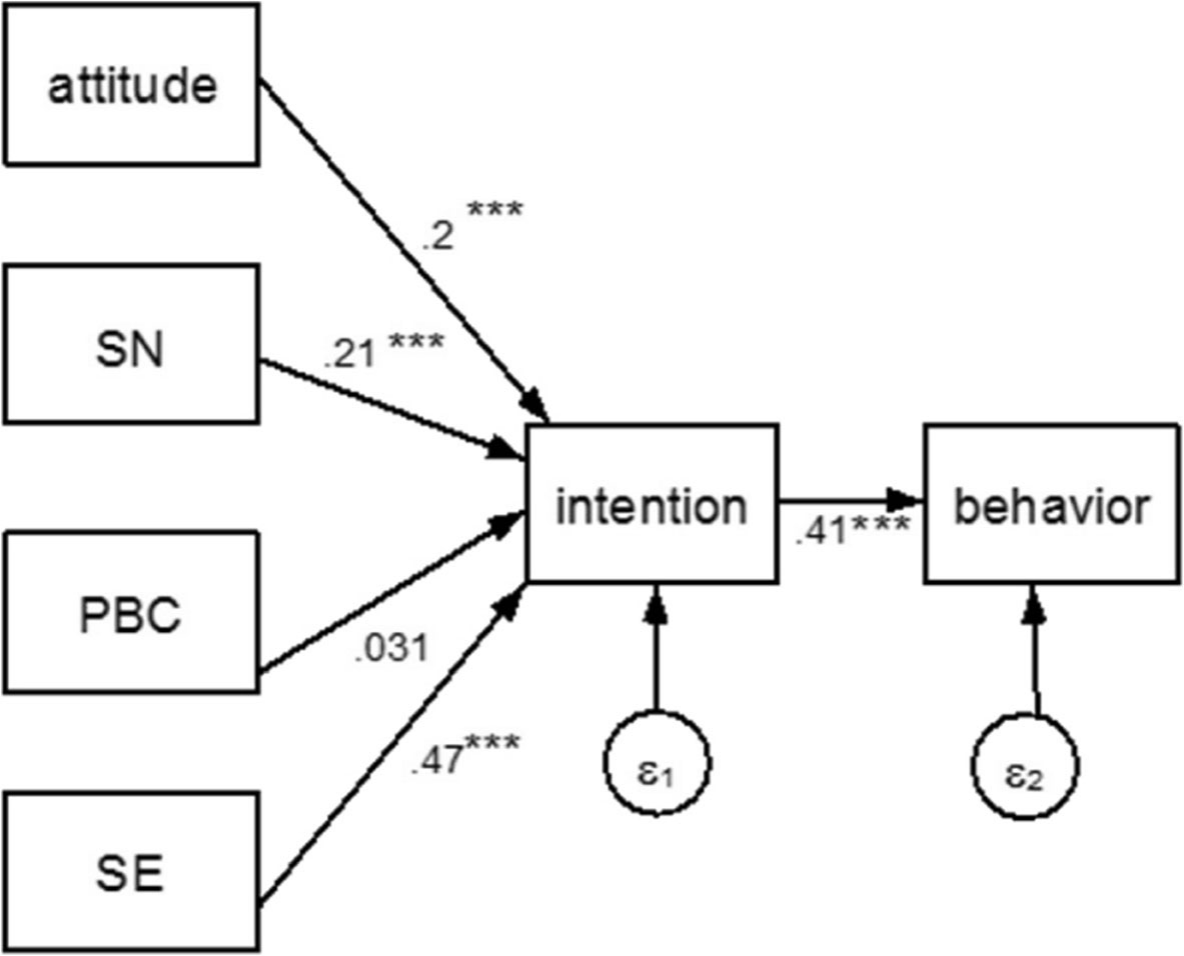
Path analysis. Notes: SN social norm, PBC perceived behavioural control, SE self-efficacy. *** *p* < 0.01

The next step was to estimate the structural equation model in the form of path analysis using maximum likelihood method. The model with standardized coefficients and *p*-values is reported in Fig. 2. Except for PBC, attitude, social norm, and self-efficacy were all statistically associated with behavioural intention (their coefficients were, respectively β_A➔*I*_ = 0.2, β_SN➔*I*_ = .21, and β_SE➔*I*_ = 0.47, *p* < 0.01). In the model’s structure, past behaviour was employed as a proxy for actual behaviour. The path connecting intention to behaviour was also high and significant (β_I➔B_ 0.41, *p* < 0.01). The model yielded, a chi-square test of 25.90 (df = 4, *p* < 0.01), CFI = 0.92 and RMSEA = 0.116 which might indicated substandard fit. In fact, in structural equation model it is expected a non-significant chi-square to claim a reasonable fit. However, as shown in the literature, if the sample size is above 200 units, as in our case, it is very common to obtain a significant chi-square, even when the model is accurate [30]. Therefore, other fit indices should be considered. The CFI is above 0.90 indicates that our model does 92% better than a null model that assumes that all the coefficients are unrelated to each other, but the RAMSEA not below the recommended 0.1.

In order to observe how each model factor affected intention, OLS stepwise regression models were successively run. As reported in Table 3, each model adds a new predictor in the specification. As long as more constructs were included in the model, the R-squared substantially improved, going from R^2^ = 0.119 in Model 1 where only attitude was included, to R^2^ = 0.416 in Model 4 were all the predictors were added. Attitude, social norm, self-efficacy, and past behaviour were all positively related to the intention of wearing a pollution mask and also highly significant (specifically, β_A_ = 0.23, *p* < 0.01, β_SN_ = 0.37, *p* < 0.01, β_SN_ = 0.32, *p* < 0.01, β_PB_ = 0.12, *p* < 0.01). PBC was instead not significantly correlated with intention.

**Table 3:** Step-wise OLS regression, beta and standard errors in parenthesis

Variables	Model 1	Model 2	Model 3	Model 4	Model 5
Attitude	0.411***(0.0630)	0.296***(0.0651)	0.303***(0.0642)	0.245***(0.0544)	0.228***(0.0611)
Social norm		0.545***(0.0866)	0.507***(0.0882)	0.344*** (0.0802)	0.375***(0.0997)
PBC			0.162*** (0.0531)	0.0236 (0.0532)	−0.0364 (0.0614)
Self-efficacy				0.380*** (0.0404)	0.316*** (0.0473)
Past-behaviour					0.116*** (0.0377)
Gender	0.210 (0.158)	0.110 (0.154)	0.0991 (0.152)	0.0802 (0.136)	0.0551 (0.148)
Residency	0.0117 (0.185)	0.115 (0.178)	0.0455 (0.177)	0.127 (0.160)	0.176 (0.169)
Age	0.0379 (0.0409)	0.0353 (0.0401)	0.0261 (0.0395)	0.0142 (0.0285)	0.0333 (0.0351)
*R-squared*	*0.119*	*0.213*	*0.238*	*0.404*	*0.416*

Controls for gender, residency, and age were also included

Notes: beta and SE; *** *p*-values < 0.01

## Discussion

With the worsening of the air quality, the Chinese population’s awareness of both the issue itself and its health consequences has rapidly increased [31]. Underperformance of risk-averse behaviour seems more acute among a human asset of crucial importance for the country: its college-level generation. This paper was intended to describe the effects of a wider set of influences beyond information exposure to understand the actual triggers which are more likely to favor the intention of wearing a pollution mask. Highly educated individuals, i.e., young college students, a segment of the population which is plausibly quite able to understand and assimilate cost-benefit analysis and efficiently conduct the rational processing of scientific information would be expected to respond positively to reason-based cognitive stimuli. Yet, social norms, self-efficacy, attitudes, and past behaviour, all clearly played a critically important role in the decision of wearing a mask.

Our findings suggest that young Chinese college students condition their use of pollution mask to what their social circle think of it, in particular friends and roommates. The social legitimacy of this action is a critical shaper of their intentions. Accordingly a language and approach that reinforces social adequacy and “coolness” of the target behaviour may prove effective in mobilizing facemask use through peer-pressure. This finding falls in line with studies showing how social pressure critically influenced mask wearing during the outbreak of SARS [32] as well as for favouring other health prevention measures especially among youngest cohorts [33] and during quarantine times [34].

An individual’s perception of his/her competence to enact the action is also critical, as epitomized by the high loading of the self-efficacy variable. Interestingly, this effect runs independently of individual perception of external obstacles or facilitators to attain the ultimate result. This finding echoes other studies analysing the role of self-efficacy in following preventive behaviours [35, 36]. Accordingly, appealing to this sense of impact of one’s confidence in implementing the behaviur is critical to scaling up facemask use. Conversely, given that wearing a pollution mask requires little volitional control, behaviour control-related barriers like price, quality, and brand recommendation hardly determines the odds of performing the target behaviour. Naturally, the practical irrelevance of TPB for facemask use cannot be extended to other health prevention behaviors. In fact, TPB has proved greatly effective in shaping early cancer screenings and use of seat-belts [37].

Our study also confirmed the importance of past behaviour which, in this case, strengthens the relationship between intention and behaviour by acting as an intention stabilizer. In general, the inclusion of past behaviour had been shown to improve the prediction of future behaviour and, also, had sometimes been found to be the only significant predictor [28].

This validates the analytical gains of putting behavioural change into a broader and more realistic context than merely knowledge exposure. It also signals the potential advantages of identifying a more effective mobilizational language to encourage a wider embracement of the proposed action. In the health promotion literature, there are several examples on how by slightly changing behavioural triggers, or by adding almost-invisible nudges, individuals are more likely going to adopt healthier behaviours [38].

### Implications and limitations

Past evidence has proven how public health programs aimed at increasing the adoption of preventive behaviour are more successful when the health intervention design includes the understanding of the complexity of the behavioural determinants [36]. The variations in individual responses to smog are also critical to the development of adequate public policies, as well as interventions to promote changes in behaviours [39, 40]. Besides government and Universities’ actions aimed at reducing pollution levels by rackling the sources of it, self-protection measures are taken at the individual level, and are not homogenous among different echelons of the society. A recent study from Zhang et al. [16] found for example that richer and urban Chinese are much more likely willing to invest in anti-pollution masks and air-filters.

Our study suggests that knowledge-intensive approaches to mobilize individuals towards new health-preventive behaviours do not warrant successful results. To succeed, government and institutional programs are required to support the adoption of new habits by framing them in a language of social legitimacy, that is, by building a narrative of the expected behaviour that socially relevant references (peers) demand from the individual or in terms of levelling up with the socially acceptable behaviour already set in practice by relevant personal references (legitimacy). Communication approaches that motivate mask-wearing in social neighbours or convoke to champion one’s social circle by meeting loved ones’ expectations to take care of one’s health may prove effective. A language around social comparisons may thus prove effective. Consequently, given the salience of social norms (particularly among educated youngsters) using celebrities, key opinion leaders, or authoritative spoke-persons to highlight the reputation gains of adopting new behaviours (or the embarrassment of not doing so) can help overcome resistance or indifference. Conversely, our results suggest that it might be pointless to emphasize exclusively accessibility of affordability issues, or execute any action on those domains, as these are not recognized as obstacles or barriers towards higher pollution mask use among students. In other terms, as these conditions are necessary, they might be confused as sufficient factors leading to the straight adoption of the intended behaviour. On the other hand, interventions aimed at altering the opportunity costs of maintaining forms of social status and self-image, as acquired from adopting or omitting the advocated behaviour, might reveal themselves more effective and successful. This has to work both on the side of self-perception and peer-effect.

This study has one limitation that needs to be acknowledged. It relates to the sample which is composed of students, not a representative cross-section of population, therefore it is not possible to stretch inferences to urban China as a whole. Having said that, given that young, highly educated urban Chinese seem to compose one of the groups more resistant to adopt this health prevention measure, it makes sense to prioritize motivations for mask use among this social segment. Arguments for and against the use of college students as research subjects have tended to focus on whether results obtained from such subjects are generalizable to non-student populations. In our case, and for the reasons reported above, this research is less subject to this critique, as it is already studying the population of interest, and is less in need to justify the external validity of our effort. Researchers such as Kardes [41] and Lucas [42] have argued that college students are appropriate research subjects when the research emphasis is on basic psychological processes or the theory tested links to human behaviours independent of sample characteristics. According to Berkowitz and Donnerstein [43], the *“meaning the subjects assign to the situation they are in and the behaviour they are carrying out plays a greater part in determining the generalizability of an experiment’s outcome than does the sample’s demographic representativeness.”* However, other researchers, such as Sears [44] and Gallander, North, and Sugar [45], have expressed unease about the use of a narrow database of college students in behavioural research. In particular, Sears suggests that what is apparently “known” about humans is biased because college students tend to have stronger cognitive skills, less crystallized attitudes, more compliant behaviour, and less stable peer group relationships than older adults. It cannot be denied that such characteristics can endanger the external validity of survey studies. However, in our case, these are all factors that help understanding better the main mechanisms, as these underlying characteristics have at least been found to be salient when talking about healthy behaviour in this framework. While the risks of over response cannot be denied, selecting individuals possessing on average stronger cognitive skills would certainly bias our findings, but in the sense that responses are *stronger* not *weaker* in the whole population, given that non-rational drivers would presumably be amplified in this second case. This might at least in part mitigate our bias.

## Conclusions

Despite the low cost, accessibility, and easiness of using pollution masks, and notwithstanding the public campaigns and media coverage stressing the associated benefits of their usage, numerous individuals struggle to adopt this kind of healthy behaviour [46]. So, if information dissemination and knowledge-based mobilization has occupied a central place in the governmental strategy to redress the negative outcomes of air pollution among individuals, net results of such attempts in terms of pollution mask use have frustrated expectations, and this happened in particular among one of the most valuable human resource assets of the nation: its college-level youth. This evidence challenges reason-based preventive medicine approaches, which are centred on pure cognitive-appraising stimuli or straightforward cost-benefit propositions which should more or less automatically lead to the adoption of the healthy behaviour. Our results depict a more complex picture of cognitive behaviour, one that encourages to go beyond awareness-raising or cognitive enticement models to explain behavioural outcomes. The role of social norms suggest the usage of social cues enhancing the behaviour acceptability and desirability, thus helping to revise perceptions of the targeted action as positively aligned with aspirational life-styles. By placing pollution mask wearing in a less defensive narrative (e.g. avoiding risk) and connecting its usage to positive traits associated with social inclusiveness or emulation of valued social references’ behaviors, young-well educated Chinese may begin to change, first, perceptions and, then, actions. The findings of this paper could help both health operators and facemask producers to improve the design of environmental health intervention campaigns, although further evidence is needed to generalize the results to a broader population.

## Additional file

Additional file 1: Survey Dataset. (DTA 80 kb)

## Abbreviations

AQI: Air Quality Index
CFI: Comparative Fix Index
DF: degree of freedom
OLS: Ordinary Least Squares
PBC: Perceived Behavioural Control
PM: particulate matter
RMSEA: Root Mean Square Error of Approximation
SE: standard errors
SN: Social Norm
TPB: Theory of Planned Behaviour

## References

[1] Kan H, Chen B, Hong C (2009). Health impact of outdoor air pollution in China: current knowledge and future research needs. Environ Health Perspect.

[2] Samoli E, Atkinson RW, Analitis A, Fuller GW, Green DC, Mudway I (2016). Associations of short-term exposure to traffic-related air pollution with cardiovascular and respiratory hospital admissions in London, UK. Occup Environ Med.

[3] Hsu, A. et al. The 2014 Environmental Performance Index. (Yale Center for Environmental Law and Policy, 2014).

[4] Aunan K, Pan X-C (2004). Exposure-response functions for health effects of ambient air pollution applicable for China–a meta-analysis. Sci Total Environ.

[5] Baccarelli A, Barretta F, Dou C, Zhang X, McCracken JP, Díaz A (2011). Effects of particulate air pollution on blood pressure in a highly exposed population in Beijing, China: a repeated-measure study. Env Health.

[6] Chen X, Zhang X, Zhang X. Smog in Our Brains: Gender Differences in the Impact of Exposure to Air Pollution on Cognitive Performance. International Food Policy Research Institute (IFPRI); 2017.

[7] Fleisch AF, Kloog I, Luttmann-Gibson H, Gold DR, Oken E, Schwartz JD (2016). Air pollution exposure and gestational diabetes mellitus among pregnant women in Massachusetts: a cohort study. Environ Health.

[8] Kan H, London SJ, Chen G, Zhang Y, Song G, Zhao N (2008). Season, sex, age, and education as modifiers of the effects of outdoor air pollution on daily mortality in Shanghai, China: the public health and air pollution in Asia (PAPA) study. Environ Health Perspect.

[9] Wang Y, Sun M, Yang X, Yuan X (2016). Public awareness and willingness to pay for tackling smog pollution in China: a case study. J Clean Prod.

[10] Joss MK, Eeftens M, Gintowt E, Kappeler R, Künzli N (2017). Time to harmonize national ambient air quality standards. Int J Public Health.

[11] Bell Matthew. Beijingers don masks to defend themselves against dirty air — and to make a fashion statement [internet]. PRI 2013. Available from: https://www.pri.org/stories/2013-11-11/beijingers-don-masks-defend-themselves-against-dirty-air-and-make-fashion

[12] Zhang S, Li L, Gao W, Wang Y, Yao X (2016). Interventions to reduce individual exposure of elderly individuals and children to haze: a review. J Thorac Dis.

[13] Smith JD, MacDougall CC, Johnstone J, Copes RA, Schwartz B, Garber GE (2016). Effectiveness of N95 respirators versus surgical masks in protecting health care workers from acute respiratory infection: a systematic review and meta-analysis. Can Med Assoc J.

[14] Witte K, Allen M (2000). A meta-analysis of fear appeals: implications for effective public health campaigns. Health Educ Behav.

[15] Maddux JE, Rogers RW (1983). Protection motivation and self-efficacy: a revised theory of fear appeals and attitude change. J Exp Soc Psychol.

[16] Zhang J, Mu Q. Air pollution and defensive expenditures: Evidence from particulate-filtering facemasks. J Environ Econ Manag. 2017; doi: 10.1016/j.jeem.2017.07.006.

[17] Ajzen I (1991). The theory of planned behavior. Organ Behav Hum Decis Process.

[18] Lin TT, Bautista JR (2016). Predicting intention to take protective measures during haze: The roles of efficacy, threat, media trust, and affective attitude. J Health Commun.

[19] Zhou G, Gan Y, Ke Q, Knoll N, Lonsdale C, Schwarzer R (2016). Avoiding exposure to air pollution by using filtering facemask respirators: an application of the health action process approach. Health Psychol.

[20] Roncancio AM, Ward KK, Sanchez IA, Cano MA, Byrd TL, Vernon SW (2015). Using the theory of planned behavior to understand cervical cancer screening among Latinas. Health Educ Behav.

[21] Juraskova I, O’Brien M, Mullan B, Bari R, Laidsaar-Powell R, McCaffery K (2012). HPV vaccination and the effect of information framing on intentions and behaviour: an application of the theory of planned behaviour and moral norm. Int J Behav Med..

[22] Glanz K, Rimer BK, Viswanath K (2008). Health behavior and health education: theory, research, and practice.

[23] Acock AC (2013). Discovering structural equation modeling using Stata.

[24] Strecher VJ, McEvoy DeVellis B, Becker MH, Rosenstock IM (1986). The role of self-efficacy in achieving health behavior change. Health Educ Q.

[25] Rhodes RE, Courneya KS (2003). Self-efficacy, controllability and intention in the theory of planned behavior: measurement redundancy or causal independence?. Psychol Health.

[26] Manstead AS, Eekelen SA (1998). Distinguishing between perceived behavioral control and self-efficacy in the domain of academic achievement intentions and behaviors. J Appl Soc Psychol.

[27] Povey R, Conner M, Sparks P, James R, Shepherd R (2000). Application of the theory of planned behaviour to two dietary behaviours: roles of perceived control and self-efficacy. Br J Health Psychol.

[28] McEachan RRC, Conner M, Taylor NJ, Lawton RJ (2011). Prospective prediction of health-related behaviours with the theory of planned behaviour: a meta-analysis. Health Psychol Rev.

[29] Comrey A, Lee H (1992). Interpretation and application of factor analytic results. Comrey AL Lee HB First Course Factor Anal.

[30] Barrett P (2007). Structural equation modelling: adjudging model fit. Personal Individ Differ.

[31] Hu L, Zhu L, Xu Y, Lyu J, Imm K, Yang L (2017). Relationship between air quality and outdoor exercise behavior in China: a novel Mobile-based study. Int J Behav Med.

[32] Tang CS, Wong C (2004). Factors influencing the wearing of facemasks to prevent the severe acute respiratory syndrome among adult Chinese in Hong Kong. Prev Med.

[33] Wong C-Y, Tang CS-K (2005). Practice of habitual and volitional health behaviors to prevent severe acute respiratory syndrome among Chinese adolescents in Hong Kong. J Adolesc Health.

[34] Cava MA, Fay KE, Beanlands HJ, McCay EA, Wignall R (2005). Risk perception and compliance with quarantine during the SARS outbreak. J Nurs Scholarsh.

[35] Bish A, Michie S (2010). Demographic and attitudinal determinants of protective behaviours during a pandemic: a review. Br J Health Psychol.

[36] Glanz K, Bishop DB (2010). The role of behavioral science theory in development and implementation of public health interventions. Annu Rev Public Health.

[37] Ojo TK (2018). Seat belt and child restraint use in a developing country metropolitan city. Accid Anal Prev.

[38] Montano DE, Kasprzyk D. The theory of reasoned action and the theory of planned behavior. Glanz, K Lewis, FM Rimer, BK eds. Health Behavior and Health Education, Theory, Research and Practice Jossy Bass San Francisco, CA. 2002. p. 85–112.

[39] Ban J, Zhou L, Zhang Y, Anderson GB, Li T (2017). The health policy implications of individual adaptive behavior responses to smog pollution in urban China. Environ Int.

[40] Wang F, Zhao H, Zhang X, Niu C, Ma J. Understanding individual-level protective responses to air pollution warning: a case study of Beijing, China. Hum Ecol Risk Assess Int J 2018;1–15.

[41] Sun C, Kahn ME, Zheng S (2017). Self-protection investment exacerbates air pollution exposure inequality in urban China. Ecol Econ.

[42] Lucas K, Sherry JL (2004). Sex differences in video game play: a communication-based explanation. Commun Res.

[43] Leonard B, Edward D (1982). External validity is more than skin deep: some answers to criticisms of laboratory experiments. Am Psychol.

[44] Sears DO (1983). The person-positivity bias. J Pers Soc Psychol.

[45] Wintre G, Maxine NC, Sugar Lorne A (2001). Psychologists’ response to criticisms about research based on undergraduate participants: a developmental perspective. Can Psychol.

[46] Şimşekoğlu Ö, Lajunen T (2008). Social psychology of seat belt use: a comparison of theory of planned behavior and health belief model. Transp Res Part F Traffic Psychol Behav.

